# Supplementary material for: Health impact and cost-effectiveness analysis of gender-neutral versus female-only 9-valent human papillomavirus vaccination in Taiwan
Source: PLoS One. 2025 Oct 14;20(10):e0333757. doi: 10.1371/journal.pone.0333757 (PMC12520334; doi:10.1371/journal.pone.0333757)
Supplement: S1 Table — (DOCX) [file pone.0333757.s001.docx]

**S1 Table. Attribution of disease to specific HPV types**

| Cancer type | ICD-10 codes | Prevalence  (% of cancers or GW) | Prevalence (% of HPV+ cancers) | Prevalence (% of HPV+ cancers) | Prevalence (% of HPV+ diseases | Reference(s) |
| --- | --- | --- | --- | --- | --- | --- |
|  |  |  | HPV16/18 | HPV16/18 + 31, 33, 45, 52 and 58 | HPV6/11 |  |
| Cervical cancer | C53 | 100.0% | 71.4% | 100.0% |  | [1] |
| Vaginal cancer | C52 | 69.3% | 38.2% | 69.6% |  | [2] |
| Vulvar cancer | C51 | 15.9% | 84.4% | 94.3% |  | [3] |
| Anal cancer | C21 | 89.0% | 79.0% | 95.0% |  | [4] |
| Oral cavity | C01-02 (tongue) | 19.4% | 67.5% | 87.1% |  | [5] |
| Oropharynx | C09 (tonsil) | 25.0% | 77.9% | 77.9% |  | [6,7] |
| Larynx | C32 | 13.2% | 85.7% | 100.0% |  | [8] |
| Penile cancer | C60 | 79.6% | 70.2% | 79.4% |  | [9] |
| Genital warts |  | 100.0% |  |  | 90.0% | [10] |

**References**

1. Chen CA, Liu CY, Chou HH, Chou CY, Ho CM, Twu NF, et al. The distribution and differential risks of human papillomavirus genotypes in cervical preinvasive lesions: A Taiwan Cooperative Oncologic Group Study. Int J Gynecol Cancer 2006;16:1801-8.

2. Chao A, Chen TC, Hsueh C, Huang CC, Yang JE, Hsueh S, et al. Human papillomavirus in vaginal intraepithelial neoplasia. Int J Cancer 2012;131:E259-68.

3. Hartwig S, St Guily JL, Dominiak-Felden G, Alemany L, de Sanjosé S. Estimation of the overall burden of cancers, precancerous lesions, and genital warts attributable to 9-valent HPV vaccine types in women and men in Europe. Infect Agent Cancer 2017;12:19.

4. Lin C, Franceschi S, Clifford GM. Human papillomavirus types from infection to cancer in the anus, according to sex and HIV status: a systematic review and meta-analysis. Lancet Infect Dis 2018;18:198-206.

5. Lee LA, Huang CG, Tsao KC, Liao CT, Kang CJ, Chang KP, et al. Human Papillomavirus Infections are Common and Predict Mortality in a Retrospective Cohort Study of Taiwanese Patients With Oral Cavity Cancer. Medicine (Baltimore) 2015;94:e2069.

6. Chen TC, Wu CT, Ko JY, Yang TL, Lou PJ, Wang CP, et al. Clinical characteristics and treatment outcome of oropharyngeal squamous cell carcinoma in an endemic betel quid region. Sci Rep 2020;10:526.

7. Liao CW. Oropharyngeal Squamous Cell Carcinoma is Associated with High-Risk Human Papillomavirus in Taiwan. AACR-AHNS: American Association for Cancer Research; 2019.

8. Chen WC, Chuang HC, Lin YT, Huang CC, Chien CY. Clinical impact of human papillomavirus in laryngeal squamous cell carcinoma: a retrospective study. PeerJ 2017;5:e3395.

9. Alemany L, Cubilla A, Halec G, Kasamatsu E, Quiros B, Masferrer E, et al. Role of Human Papillomavirus in Penile Carcinomas Worldwide. Eur Urol 2016;69:953-61.

10. Tsai TF, Kothari-Talwar S, Yee K, Kulkarni A, Lara N, Roset M, et al. Estimating the burden of genital warts in Taiwan. Sex Health 2017;14:485-91.
