# Supplementary material for: Health impact and cost-effectiveness analysis of gender-neutral versus female-only 9-valent human papillomavirus vaccination in Taiwan
Source: PLoS One. 2025 Oct 14;20(10):e0333757. doi: 10.1371/journal.pone.0333757 (PMC12520334; doi:10.1371/journal.pone.0333757)
Supplement: S2 Table — (DOCX) [file pone.0333757.s002.docx]

**S2 Table. Inputs for the demographic module: population counts, annual mortality, and sexual behavior**

| Input parameter | Males | Females | Source |
| --- | --- | --- | --- |
| National population (year: 2017) | 11,719,580 (49.72%) | 11,851,647 (50.28%) | Ministry of the Interior, Taiwan [1] |
| Annual all-cause mortality rates for the general population by age group, per capita |  |  | Ministry of the Interior, Taiwan [1] |
| 0-4 years | 0.0009 | 0.0009 |  |
| 5-9 years | 0.0001 | 0.0001 |  |
| 10–14 years | 0.0002 | 0.0001 |  |
| 15–19 years | 0.0005 | 0.0002 |  |
| 20-24 years | 0.0006 | 0.0003 |  |
| 25-29 years | 0.0006 | 0.0003 |  |
| 30–34 years | 0.0011 | 0.0005 |  |
| 35–39 years | 0.0018 | 0.0007 |  |
| 40–44 years | 0.0030 | 0.0011 |  |
| 45-49 years | 0.0045 | 0.0016 |  |
| 50-54 years | 0.0065 | 0.0024 |  |
| 55–59 years | 0.0087 | 0.0035 |  |
| 60–64 years | 0.0118 | 0.0051 |  |
| 65–69 years | 0.0165 | 0.0079 |  |
| 70-74 years | 0.0271 | 0.0150 |  |
| 75-79 years | 0.0446 | 0.0271 |  |
| 80-84 years | 0.0758 | 0.0501 |  |
| ≥85 years | 0.1244 | 0.0933 |  |
| Sexual activity category, % |  |  | Huang *et al.* (2010) [2] |
| Low (mean number of sexual partners per year: 0-1) | 85.1 | 90.7 |  |
| Medium (mean number of sexual partners per year: 2-4) | 11.9 | 7.6 |  |
| High (mean number of sexual partners per year: ≥5) | 3 | 1.7 |  |

**References**

1. Department of Household Registration, Ministry of the Interior, Republic of China (Taiwan). Statistics: Department of Household Registration, Ministry of the Interior, Republic of China (Taiwan); [November 28, 2022]. Available from: <https://www.ris.gov.tw/app/en/3910>.

2. Huang S, Sixian L, Yunzhi Z. An analysis of human behavior in Taiwan at the turn of the century: The intersection of generation, gender, education, and marital status [in Chinese]. Taiwan Journal of Sexology 2010;16:1-28.
