# Supplementary material for: Health impact and cost-effectiveness analysis of gender-neutral versus female-only 9-valent human papillomavirus vaccination in Taiwan
Source: PLoS One. 2025 Oct 14;20(10):e0333757. doi: 10.1371/journal.pone.0333757 (PMC12520334; doi:10.1371/journal.pone.0333757)
Supplement: S3 Table — (DOCX) [file pone.0333757.s003.docx]

**S3 Table. Epidemiologic input parameters: screening and treatment patterns**

| Input parameter | Males | Females | Source |
| --- | --- | --- | --- |
| Screening patterns (cervical cancer) |  |  |  |
| Females receiving a follow-up screening test after abnormal PAP smear diagnosis | -- | 88% | Cervical Cancer Screening Registry’s 2018 Annual Report [1] |
| Females receiving gynecological cancer screening tests at least once in their lifetime | -- | 88% | Cervical Cancer Screening Registry’s 2018 Annual Report [1] |
| Females screened for cervical cancer in the past year, by age group |  |  | Cervical Cancer Screening Registry’s 2018 Annual Report [1] |
| 0-19 | -- | 0% |  |
| 20-24 | -- | 2.50% |  |
| 25-26 | -- | 6.60% |  |
| 27-29 | -- | 6.60% |  |
| 30-34 | -- | 27.10% |  |
| 35-39 | -- | 29.30% |  |
| 40-44 | -- | 29.50% |  |
| 45-49 | -- | 32.90% |  |
| 50-54 | -- | 31.40% |  |
| 55-59 | -- | 29.50% |  |
| 60-64 | -- | 27.90% |  |
| 65-69 | -- | 27.00% |  |
| ≥70 | -- | 9.90% |  |
| PAP screening specificity value | -- | 98.6% | Chao *et al.* (2008) [2] |
| Treatment patterns |  |  |  |
| Female population receiving hysterectomy over the course of 1 year by age group, % |  |  | NHIRD inpatient claims 2015-2016 [3] |
| <1 | -- | 0.0000% |  |
| 1-8 | -- | 0.0000% |  |
| 9-10 | -- | 0.0000% |  |
| 11-12 | -- | 0.0000% |  |
| 13-14 | -- | 0.0000% |  |
| 15-17 | -- | 0.0002% |  |
| 18 | -- | 0.0000% |  |
| 19 | -- | 0.0000% |  |
| 20-24 | -- | 0.0001% |  |
| 25-26 | -- | 0.0010% |  |
| 27-29 | -- | 0.0032% |  |
| 30-34 | -- | 0.0048% |  |
| 35-39 | -- | 0.0077% |  |
| 40-44 | -- | 0.0145% |  |
| 45-49 | -- | 0.0185% |  |
| 50-54 | -- | 0.0172% |  |
| 55-59 | -- | 0.0203% |  |
| 60-64 | -- | 0.0225% |  |
| 65-69 | -- | 0.0198% |  |
| 70-74 | -- | 0.0105% |  |
| 75-79 | -- | 0.0118% |  |
| 80-84 | -- | 0.0072% |  |
| ≥85 | -- | 0.0028% |  |

**References**

1. Health Promotion Administration, Ministry of Health and Welfare. Cervical Cancer Screening Registry System Annual Report, Republic of China, 2018 [November 28, 2022]. Available from: <https://www.hpa.gov.tw/Pages/List.aspx?nodeid=1322>.

2. Chao A, Hsu KH, Lai CH, Huang HJ, Hsueh S, Lin SR, et al. Cervical cancer screening program integrating Pap smear and HPV DNA testing: a population-based study. Int J Cancer 2008;122:2835-41.

3. National Health Insurance Research Database, Taiwan [November 29, 2022]. Available from: <http://nhird.nhri.org.tw/en/index.htm>.
