# Supplementary material for: Health impact and cost-effectiveness analysis of gender-neutral versus female-only 9-valent human papillomavirus vaccination in Taiwan
Source: PLoS One. 2025 Oct 14;20(10):e0333757. doi: 10.1371/journal.pone.0333757 (PMC12520334; doi:10.1371/journal.pone.0333757)
Supplement: S4 Table — (DOCX) [file pone.0333757.s004.docx]

**S4 Table. Economic input parameters: costs of vaccination, screening, and treatment**

| Input parameter | Cost (NTD) | Source |
| --- | --- | --- |
| Cost of vaccination |  | Health Promotion Administration [1] |
| Vaccine, per dose | 1,880 |  |
| Administration, per dose | 100 |  |
| Cost of screening and diagnosis of cervical and vaginal cancers |  |  |
| Office visit | 230 | http://sc-dr.tw/health_form/inquire/06_01.pdf |
| Colposcopy | 605 | National Health Insurance Research Database (NHIRD) [2] |
| Biopsy | 2,171 | National Health Insurance Research Database (NHIRD) [2] |
| Cost per episode of care |  |  |
| Females |  | 2014-2015 NHIRD ambulatory care claims, inpatient claims [2] |
| CIN 1 | 55,269 |  |
| CIN 2 | 55,269 |  |
| CIN 3, CIS | 55,269 |  |
| Cervical cancer |  |  |
| Local disease | 262,376 |  |
| Regional disease | 484,146 |  |
| Distant disease | 1,105,942 |  |
| Vaginal cancer |  |  |
| Local disease | 258,928 |  |
| Regional disease | 308,199 |  |
| Distant disease | 787,483 |  |
| Vulvar cancer |  |  |
| Local disease | 200,345 |  |
| Regional disease | 510,713 |  |
| Distant disease | 1,479,971 |  |
| Anal cancer |  |  |
| Local disease | 274,388 |  |
| Regional disease | 470,660 |  |
| Distant disease | 704,284 |  |
| Head & neck |  |  |
| Local disease | 222,338 |  |
| Regional disease | 461,351 |  |
| Distant disease | 711,259 |  |
| Genital warts | 2,175 |  |
| Recurrent respiratory papillomatosis | 50,716 |  |
|  |  |  |
| Males |  | 2014-2015 NHIRD ambulatory care claims, inpatient claims [2] |
| Penile cancer |  |  |
| Local disease | 109,271 |  |
| Regional disease | 417,984 |  |
| Distant disease | 1,159,698 |  |
| Anal cancer |  |  |
| Local disease | 262,228 |  |
| Regional disease | 472,207 |  |
| Distant disease | 604,349 |  |
| Head & neck cancer |  |  |
| Local disease | 266,022 |  |
| Regional disease | 515,441 |  |
| Distant disease | 905,908 |  |
| Genital warts | 2,763 |  |
| Recurrent respiratory papillomatosis | 286,056 |  |

CIN, cervical intraepithelial neoplasia; CIS, carcinoma in situ; NHIRD, National Health Insurance Research Database

**References**

1. Health Promotion Administration. [November 28, 2022]. Available from: <https://www.hpa.gov.tw/Pages/ashx/File.ashx?FilePath=~/File/Attach/16056/File_19682.pdf>.

2. National Health Insurance Research Database, Taiwan [November 29, 2022]. Available from: <http://nhird.nhri.org.tw/en/index.htm>.
