# Supplementary material for: Health impact and cost-effectiveness analysis of gender-neutral versus female-only 9-valent human papillomavirus vaccination in Taiwan
Source: PLoS One. 2025 Oct 14;20(10):e0333757. doi: 10.1371/journal.pone.0333757 (PMC12520334; doi:10.1371/journal.pone.0333757)
Supplement: S5 Table — (DOCX) [file pone.0333757.s005.docx]

**S5 Table. Calibration results**

|  |  | **Incidence** | **Mortality** |  | **Incidence** | **Mortality** |
| --- | --- | --- | --- | --- | --- | --- |
|  |  | **4vHPV** | **4vHPV** |  | **9vHPV** | **9vHPV** |
| Cervical cancer |  |  |  |  |  |  |
|  | Observed | 7.24 | 4.05 |  | 10.14 | 5.67 |
|  | Calibration | 7.24 | 4.09 |  | 10.14 | 5.61 |
| Vaginal cancer |  |  |  |  |  |  |
|  | Observed | 0.02 | 0.01 |  |  |  |
|  | Calibration | 0.02 | 0.01 |  |  |  |
| Vulvar cancer |  |  |  |  |  |  |
|  | Observed | 0.04 | 0.02 |  |  |  |
|  | Calibration | 0.04 | 0.02 |  |  |  |
| Anal cancer, females |  |  |  |  |  |  |
|  | Observed | 0.3 | 0.11 |  | 0.36 | 0.13 |
|  | Calibration | 0.31 | 0.11 |  | 0.36 | 0.14 |
| Anal cancer, males |  |  |  |  |  |  |
|  | Observed | 0.24 | 0.15 |  | 0.29 | 0.18 |
|  | Calibration | 0.24 | 0.15 |  | 0.27 | 0.18 |
| Head and neck cancer, females |  |  |  |  |  |  |
|  | Observed | 0.57 | 0.26 |  | 0.71 | 0.33 |
|  | Calibration | 0.57 | 0.26 |  | 0.72 | 0.33 |
| Head and neck cancer, males |  |  |  |  |  |  |
|  | Observed | 5.6 | 3.98 |  | 6.81 | 4.84 |
|  | Calibration | 5.61 | 3.98 |  | 6.76 | 4.81 |
| Penile cancer, males |  |  |  |  |  |  |
|  | Observed | 0.07 | 0.04 |  | 0.08 | 0.05 |
|  | Calibration | 0.07 | 0.04 |  | 0.08 | 0.05 |
| Genital warts, females ^A^ |  |  |  |  |  |  |
|  | Observed | 98.6 |  |  |  |  |
|  | Calibration | 98.5 |  |  |  |  |
| Genital warts, males ^A^ |  |  |  |  |  |  |
|  | Observed | 90.4 |  |  |  |  |
|  | Calibration | 90.5 |  |  |  |  |

^A^ Genital warts data are presented for HPV 6 and HPV 11.
